# Case Report: Acute Supraglottitis

**DOI:** 10.21980/J8006V

**Published:** 2020-01-15

**Authors:** Jamie Robin Chu, Jonathan G Rogg

**Affiliations:** * Department of Pediatrics-Emergency Medicine, Baylor College of Medicine, Houston, TX; ^ Department of Emergency Medicine, McGovern Medical School, Houston, TX

## Abstract

This report illustrates a case of acute supraglottitis, a rare condition that can be easily diagnosed, but can quickly be deadly if missed. The patient presented with a sore throat and unilateral neck swelling that progressed to voice changes and difficulty handling secretions. Initially, radiographs were obtained followed by computed tomography. Imaging raised concern for supraglottitis; therefore otolaryngology consult was obtained and flexible laryngoscopy confirmed the diagnosis. The patient received a prophylactic awake fiberoptic intubation at the bedside and was started on intravenous antibiotics and steroids. She quickly improved and is expected to make a full recovery. Trainees and current practicing physicians in today’s post-*Haemophilus influenza* type b vaccination period rarely encounter epiglottitis or supraglottitis. This case reminds us to keep this uncommon but quickly progressive and dangerous condition in mind to avoid a missed diagnosis which can lead to significant morbidity and mortality.

**Topics:**

Supraglottitis, acute supraglottitis, adult, intubation, antibiotic, microbiology.

[Fig f1-jetem-5-1-v12] [Fig f2-jetem-5-1-v12]

**Figure f1-jetem-5-1-v12:**
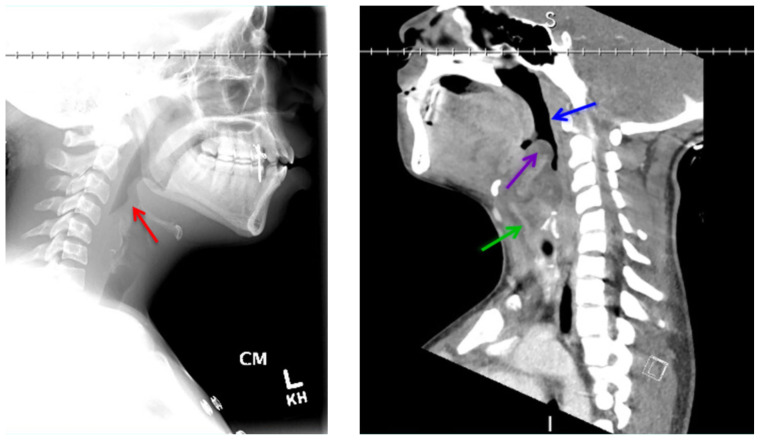


**Figure f2-jetem-5-1-v12:**
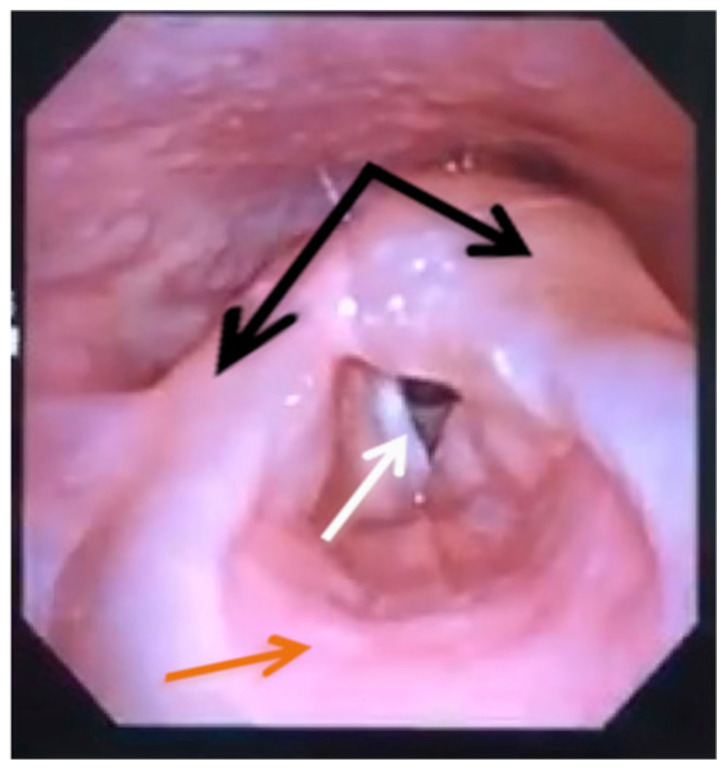


## Introduction

Acute supraglottitis is an uncommon but quickly fatal condition. The typical presentation, workup, and therapies are presented in this case. There are scattered case reports and one larger case series of supraglottitis in the literature, and many discuss delayed diagnosis as a cause of patient morbidity.^1^,^2^,^3^. This case contributes to the small fund of medical knowledge currently known on this rare condition, in the hopes of aiding quick diagnosis and treatment. This case took place in a large academic referral center with quick access to imaging and otolaryngologists. Especially for those who work in smaller community sites, early recognition of this condition with rapid coordination of resources and care could improve the patient’s outcome significantly.

## Presenting concerns and clinical findings

A 22-year-old fully vaccinated African American female with a past medical history of bipolar disorder, anxiety, cholecystectomy two years ago, and dilation and curettage for an unwanted pregnancy one-month prior presented with throat pain. The patient first developed a sore throat as well as a hoarse voice and left sided neck swelling four days prior. She was seen at an outside hospital and discharged home with a diagnosis of viral upper respiratory infection. The patient today complains of worsening throat pain and neck swelling, ongoing hoarseness, and is now having difficulty swallowing her saliva.

On arrival to the emergency department the patient’s temperature was 98.4**°**F, heart rate was 60 beats per minute, blood pressure was 111/73, respiratory rate was 18 breaths per minute, and her oxygen saturation was 100% on room air. Her voice was markedly hoarse with mild inspiratory stridor. She was spitting into a plastic bag due to inability to swallow. She otherwise was breathing comfortably with no signs of respiratory distress. Her posterior oropharynx was mildly erythematous with no exudates visualized. She had no physical manifestations of strangulation to her neck. She had significant left-sided tender submandibular lymphadenopathy. The remainder of her physical exam was normal.

## Significant findings

On arrival, radiographs of the neck soft tissues were obtained, which showed a markedly enlarged epiglottic shadow (red arrow) concerning for epiglottitis. A computed tomography scan of the neck soft tissues with contrast was then obtained which revealed edematous mucosal thickening of the oropharynx (blue arrow) and supraglottic larynx (green arrow) including the epiglottis (purple arrow) concerning for acute infectious pharyngitis and supraglottic laryngitis with severe narrowing of the supraglottic laryngeal lumen, as well as associated extensive inflammation and edema of the superficial and deep left neck spaces. The patient’s white blood cell count was elevated to 25.7×10^9^/L with 87% neutrophils. Her rapid strep test was positive. Otolaryngology was consulted and performed a bedside flexible laryngoscopy which showed significant edema of the epiglottis (orange arrow), vocal cords (white arrow), and arytenoids (black arrow), left greater than right. Based on the findings and concern for impending respiratory failure, the patient received an awake fiberoptic intubation by anesthesia at the bedside.

## Patient course

While admitted to the hospital the patient received intravenous (IV) Amoxicillin-clavulanate 3 mg every six hours and IV dexamethasone 8 mg every eight hours. She was found to have quick clinical improvement, which is commonly seen in supraglottitis once appropriate therapy is initiated.^4^ She was extubated on hospital day three and discharged home on hospital day five to complete a course of oral Amoxicillin-clavulanate 875 mg every twelve hours. Follow up one week after discharge, the patient reported mild residual sore throat, and was otherwise doing well.

## Discussion

In the post-*Haemophilus influenza* (H. flu) type b vaccine era, epiglottitis has become quite rare and is thus often misdiagnosed and mismanaged.^5^ Even rarer is supraglottitis – inflammation of the structures surrounding the epiglottis, with an incidence of 0.97–1.8 per 100,000; however, the incidence in adults has risen over the past several years.^6^ The mortality of supraglottitis is as high as 20% due to upper airway obstruction.^6^ Initial presenting symptoms are sore throat and odynophagia, followed by hoarseness or muffled voice, drooling, stridor, and possible tenderness over the anterior neck.^2^,^3^ Anxiety due to the sensation of upper airway obstruction can worsen the symptoms. A number of organisms have been implicated from pharyngeal sampling, the most common being H. flu and beta-hemolytic-streptococcus (the likely etiology in our patient), *Streptococcus pneumonia*, *Staphylococcus aureus* (including methicillin-resistant strains), and *Moraxella catarrhalis*.^5^ Viral etiologies are possible as well. Empiric antibiotic therapy is recommended with a thirdgeneration cephalosporin (such as ceftriaxone) and an antistaphylococcal agent (such as clindamycin if the patient does not appear septic, or vancomycin if there is a geographic prevalence of clindamycin-resistant strains and/or there is concern for sepsis).^6^ Obtain a blood culture prior to starting antibiotics if possible. There is no evidence showing direct benefit from glucocorticoid administration; however, steroids remain commonly used in this patient population, and studies have also failed to show adverse effects.^7^ Prophylactic intubation is favored by some providers given the unpredictable nature of the disease process.^8^ If attempted, intubation should be performed by an experienced individual because further airway trauma can exacerbate edema, lead to hemorrhage, and the need for a tracheostomy.^5^

Acute supraglottitis is a rare but dangerous condition that can have presenting symptoms similar to more benign diagnoses such as a viral upper respiratory infection. Especially in the patient with sore throat and intolerance of secretions, emergency providers should keep in mind the possibility of this condition to avoid a missed diagnosis.

## Supplementary Information












