# Supplementary figures and images for: Case Report: Acute Supraglottitis
Source: J Educ Teach Emerg Med. 2020 Jan 15;5(1):V12–4. doi: 10.21980/J8006V (PMC10332529; doi:10.21980/J8006V)

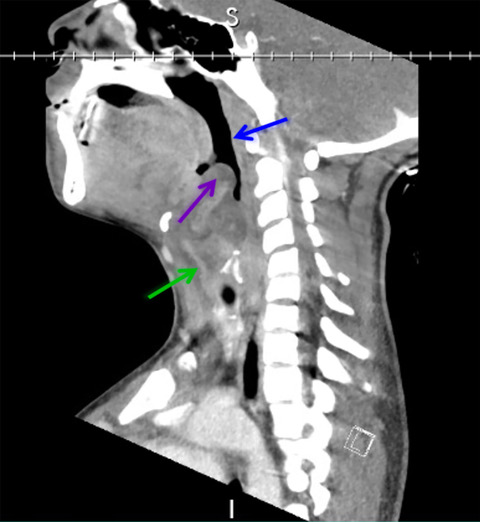

Supplement: Supplementary file 1 [file jetem-5-1-v12-supp1.jpeg]

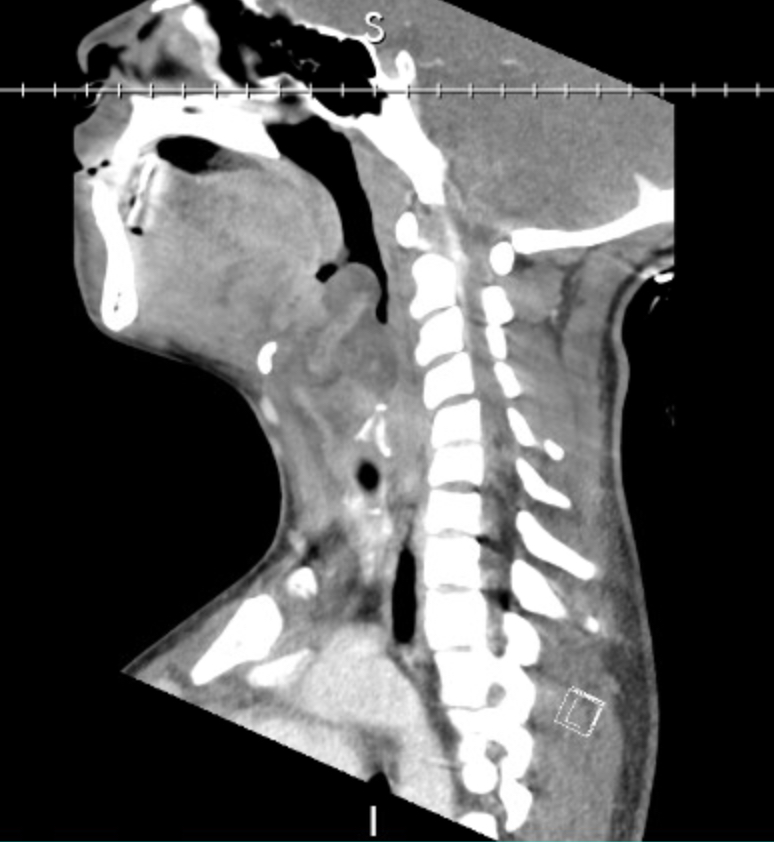

Supplement: Supplementary file 2 [file jetem-5-1-v12-supp2.jpeg]

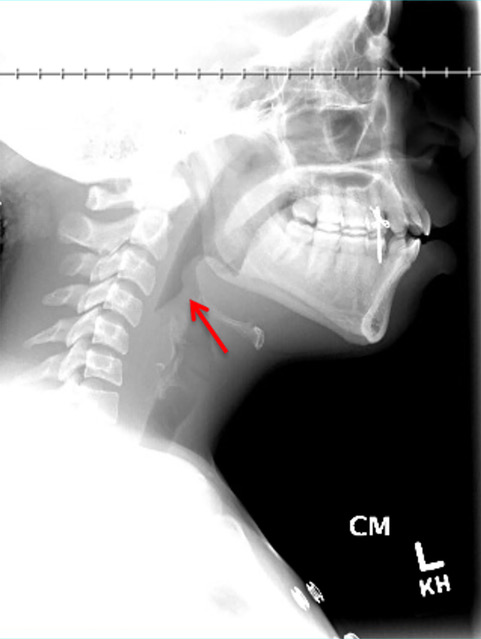

Supplement: Supplementary file 3 [file jetem-5-1-v12-supp3.jpeg]

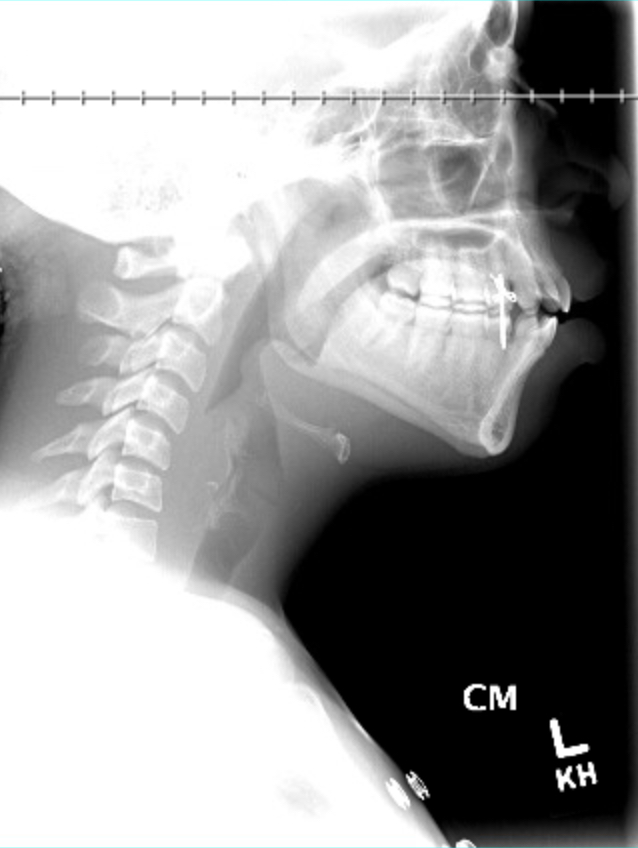

Supplement: Supplementary file 4 [file jetem-5-1-v12-supp4.jpeg]

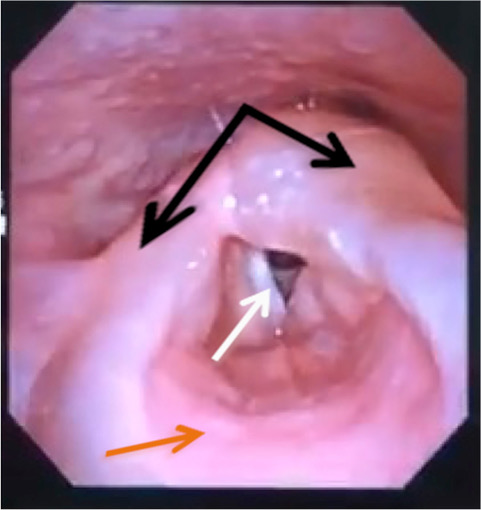

Supplement: Supplementary file 5 [file jetem-5-1-v12-supp5.jpeg]

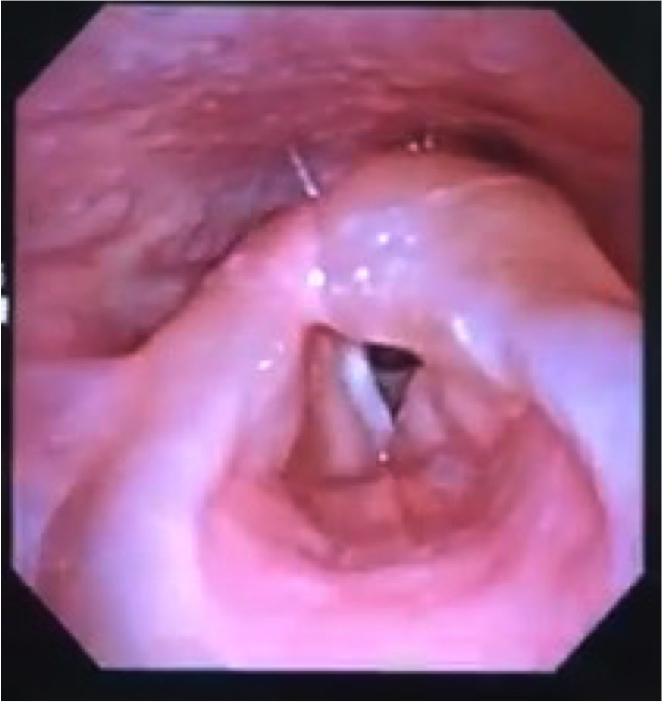

Supplement: Supplementary file 6 [file jetem-5-1-v12-supp6.jpeg]
